# Chemical Markers of Human Tendon Health Identified Using Raman Spectroscopy: Potential for In Vivo Assessment

**DOI:** 10.3390/ijms232314854

**Published:** 2022-11-27

**Authors:** Nai-Hao Yin, Anthony W. Parker, Pavel Matousek, Helen L. Birch

**Affiliations:** 1 Department of Orthopaedics and Musculoskeletal Science, University College London, UCL Stanmore Campus, RNOH, Brockley Hill, London HA7 4LP, UK; 2 Central Laser Facility, Research Complex at Harwell, Science and Technology Facilities Council, Rutherford Appleton Laboratory, UKRI, Harwell Campus, Didcot OX11 0QX, UK

**Keywords:** Achilles tendon, Raman spectroscopy, advanced glycation end-product, extracellular matrix, collagen crosslinks

## Abstract

The purpose of this study is to determine whether age-related changes to tendon matrix molecules can be detected using Raman spectroscopy. Raman spectra were collected from human Achilles (n = 8) and tibialis anterior (n = 8) tendon tissue excised from young (17 ± 3 years) and old (72 ± 7 years) age groups. Normalised Raman spectra underwent principal component analysis (PCA), to objectively identify differences between age groups and tendon types. Certain Raman band intensities were correlated with levels of advanced glycation end-product (AGE) collagen crosslinks, quantified using conventional destructive biochemistry techniques. Achilles and tibialis anterior tendons in the old age group demonstrated significantly higher overall Raman intensities and fluorescence levels compared to young tendons. PCA was able to distinguish young and old age groups and different tendon types. Raman intensities differed significantly for several bands, including those previously associated with AGE crosslinks, where a significant positive correlation with biochemical measures was demonstrated. Differences in Raman spectra between old and young tendon tissue and correlation with AGE crosslinks provides the basis for quantifying age-related chemical modifications to tendon matrix molecules in intact tissue. Our results suggest that Raman spectroscopy may provide a powerful tool to assess tendon health and vitality in the future.

## 1. Introduction

The accumulation of non-enzymatic glycation crosslinks in collagen-rich tissues is considered one of the potential biomarkers for future clinical diagnosis and classification of musculoskeletal tissue ageing and pathology [1,2,3]. Two commonly studied glycation crosslinks in musculoskeletal tissues are pentosidine and glucosepane, which are positively correlated with tissue donor age [4,5]. Glucosepane has a greater quantity than any other advanced glycation end-product (AGE) crosslink in aged collagen-rich tissues and can disrupt the local collagen triple helix structure, increase the water content within and between collagen fibrils, and alter the thermo-kinetic properties of aged tendons [6]. Glucosepane is therefore likely to contribute a greater physiological and/or mechanical impact than other glycation crosslinks. These age-related, slowly accumulating extracellular matrix changes are subtle and asymptomatic, and, due to the nature of the glycation crosslinks, it is difficult to non-destructively or non-invasively measure these crosslinks in the clinical settings, hindering the ability to screen people and recognise early signs of tendon disease.

Raman spectroscopy measures the inelastic scattering of photons from molecules. Incident laser light induces vibrational excitation within the molecule by energy exchange between the incident photons and the molecule, causing a frequency shift between the incident and scattered photons [7]. Raman spectroscopy measures the frequency shift of the scattered photons and this correlates to the chemical’s molecular bonds, molecular structure, and polarisation. The frequency shift (plotted on the x-axis) between the incident and scattering photons, expressed in wavenumbers (cm^−1^), is independent of incident light wavelength. Under specific conditions the intensity (the y-axis) of Raman bands is proportional to the concentration of molecules interrogated. Hence, by analysing the frequency shift and the different band intensities, the molecular structure and quantitative analysis can be achieved. In essence Raman spectroscopy provides a chemical ‘fingerprint’ of the studied sample [8,9].

The signal from collagen, the primary constituent of the extracellular matrix, dominates the collected Raman spectra of skin [10], bone [11], tendon [12], and cartilage tissues [13,14] (and review [15]). Collagen produces several characteristic bands related to its composition and structure. Various strong bands at wavenumbers 800 cm^−1^ to 1000 cm^−1^ are assigned to collagen C-C backbone, proline, and hydroxyproline, the two most abundant amino acids after glycine [13,15]. Prominent amide III (centre at ~1240 cm^−1^) and amide I (centre at ~1660 cm^−1^) bands are two large multicomponent bands closely related to the collagen secondary structure [16] or the collagen fibre orientation [17]. The CH_2_/CH_3_ bending band (~1450 cm^−1^) is frequently used to indicate the total protein content or the lipid content within biological samples (see review [18]). The spectral features of collagen can be influenced by various conditions, both physiological (tissue hydration, temperature) or pathological (wound, ageing, diseases) (see review [19]). Previous work has shown that, compared to younger tendons, aged tendon spectra demonstrate higher carbohydrate-related bands (1000 to 1100 cm^−1^) [20], lower CH_2_/CH_3_ and amide I bands [17,21], or an altered polarisation index [17,22].

Raman spectroscopy is considered to have high potential as a clinical tool to detect pathological or age-related tissue compositional changes. The benefits include rapid, label-free, safe assessments (see review [13,15]), with the additional possibility to measure non-invasively through the skin [23,24,25]. Compared to bone or articular cartilage, most injury-prone tendons are relatively superficial and could be the first clinical target of Raman spectroscopy application in vivo in the rheumatology and musculoskeletal fields. To the best of our knowledge, the human Achilles tendon and tibialis anterior tendon, a pair of tendons controlling different ankle movements and serving distinct locomotion functions, have not been studied by Raman spectroscopy. Establishing the spectral baseline from ex vivo tissues represents a much needed first step towards developing Raman spectroscopy for in vivo characterisation of tendon composition.

This study aims to explore whether Raman spectroscopy can differentiate human Achilles and tibialis anterior tendon samples of different ages and whether these Raman spectral features correlate with the quantity of glycation crosslinks, measured by conventional biochemical techniques.

## 2. Results

**Figure 1:**
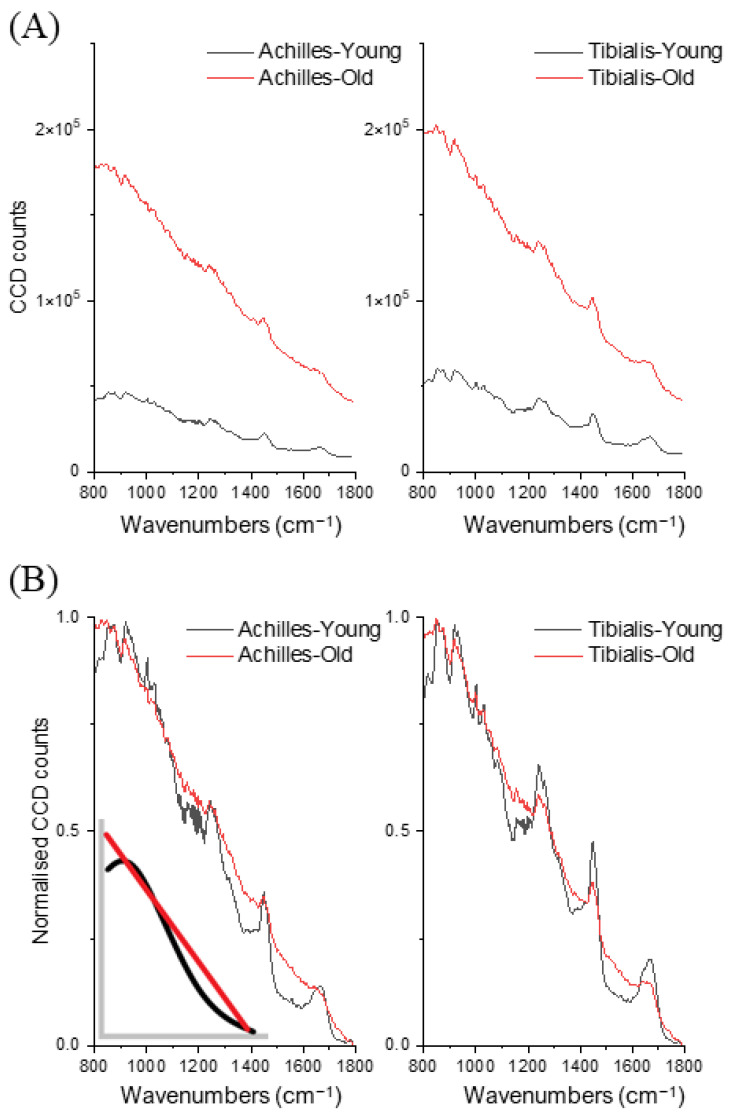
Averaged (**A**) and normalised (**B**) raw CCD counts (n = 160, 40 per group) between young and old Achilles tendons and tibialis anterior tendons. Inset: representative figure of different background shapes between young (black) and old (red) tendons.

In total 160 spectra (10 for each specimen; young:old = 80:80) were collected and analysed. The maximum of collected raw charge-coupled device (CCD) counts, which included both fluorescence background and Raman spectra, showed a more than three-times increase in the aged tendons (Figure 1A). After min-max normalisation of the fluorescence-Raman spectra, visual inspection discovered a large spectral shape difference in the fluorescence background, especially the wavenumber region between 1100 cm^−1^ and 1800 cm^−1^, where aged tendons demonstrated a higher intensity and more linear background shape than the lower intensity, reversed S-shape background of the younger tendons (Figure 1B and inset). For both tendons, the integral of raw CCD counts (excitation 830 nm) was positively correlated with collagen-linked fluorescence intensity (excitation 348 nm) measured by conventional biochemistry (Achilles: *r* = 0.67, tibialis: *r* = 0.77). Collagen-linked fluorescence in aged tendons was significantly higher than in younger tendons (Achilles: 2505 ± 262 vs. 912 ± 437, *p* < 0.001; tibialis: 1126 ± 73 vs. 271 ± 49, *p* < 0.001. Unit: relative units/mg dry weight tissue).

**Figure 2:**
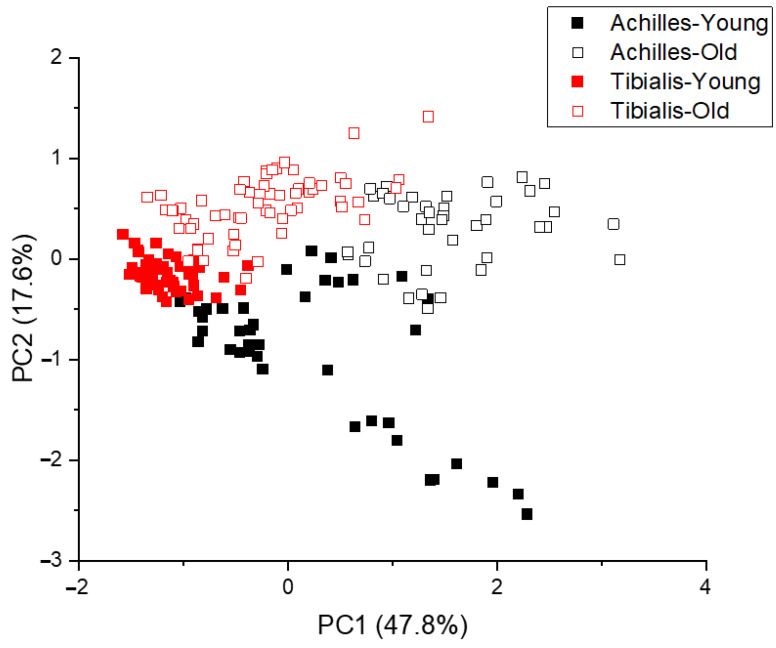
Principle component analysis of pooled Raman spectra (n = 160).

After fluorescence background removal, the underlying Raman spectra were pooled and underwent principal component analysis (PCA). The Achilles tendon and tibialis anterior tendon were separated primarily along the maximal variance axis (47.8% of variance), PC1, while the different age groups were separated along the PC2 axis (17.6% of variance), see Figure 2.

**Figure 3:**
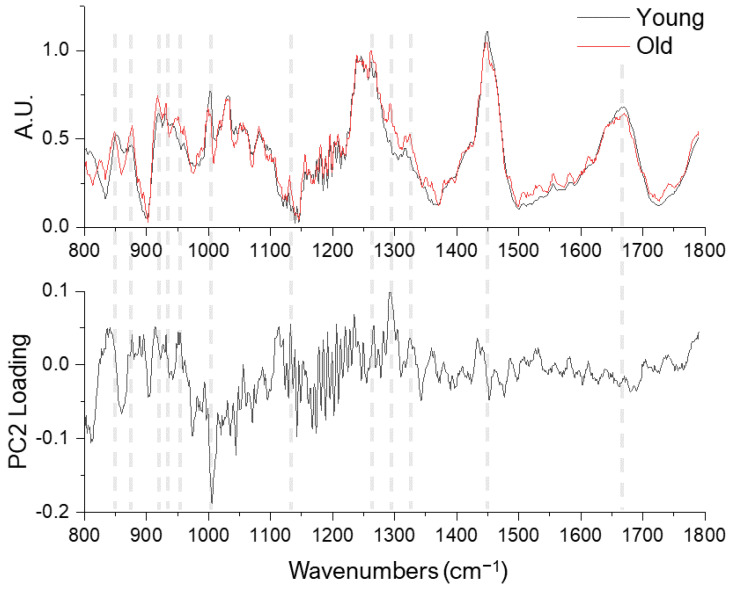
Averaged Raman spectra of young (n = 80, black lines) and old (n = 80, red lines) tendons (upper row) and the PC2 loading (lower row).

The most prominent age-related differences identified by PC2 are the 1002 cm^−1^ (phenylalanine) and 1293 cm^−1^ (amide III shoulder) bands (Figure 3). Comparing the PC2 loading and the mean Raman spectra, see Figure 3, further revealed several band intensity differences at the proline/hydroxyproline region (850, 877, 918, 931, and 951 cm^−1^), amide III and shoulder regions (1270 and 1324 cm^−1^), CH_2_ bending (1450 cm^−1^) and amide I bands (1600–1700 cm^−1^).

**Figure 4:**
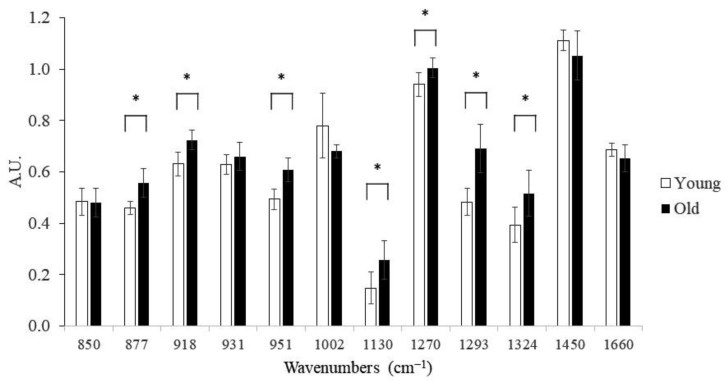
Group comparison between different Raman band intensities. Young group: n = 8 specimens, 10 spectra per specimen; Old group: n = 8 specimens, 10 spectra per specimen; *: *p* < 0.05.

Most of the band intensities, identified by PC2 loading and spectra means, were significantly different between the young and old groups (Figure 4). In addition, three bands (877, 1130, 1324 cm^−1^) that have been previously assigned to pentosidine and glucosepane [26,27] were all positively correlated with levels of these AGE crosslinks quantified using biochemical techniques (Table 1).

**Figure 5:**
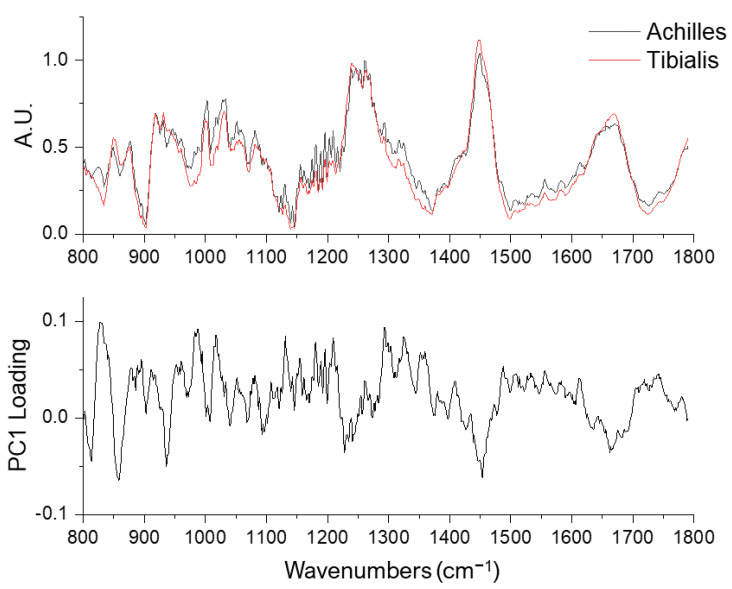
Averaged Raman spectra of Achilles (n = 80, black lines, upper row) and tibialis anterior tendons (n = 80, red lines) and the PC1 loading (lower row).

PC1 revealed compositional differences between the Achilles and tibialis anterior tendons (Figure 5 and Table 2). These differences were reflected in the proline/hydroxyproline region (800–1000 cm^−1^), carbohydrate-related bands (1000–1100 cm^−1^), the shoulder regions of amide III (~1200 cm^−1^ and ~1300 cm^−1^), CH_2_ bending band (1450 cm^−1^), and the peak intensity and the spectral shape of amide I band (1600–1700 cm^−1^). These spectral differences (identified by PC1) were not identical to the age-related changes (by PC2), demonstrating the ability to differentiate age-related and composition-related tendon molecular differences using a combination of Raman spectroscopy and principal component analysis.

## 3. Discussion

Our results demonstrate that combining Raman spectroscopy and PCA can differentiate between human Achilles tendon and tibialis anterior tendon tissue. Furthermore, Raman spectroscopy and PCA can separate both tendon types based on donor age, as previously shown for equine tendon [20]. The age-related spectral differences are highly correlated with damaging glycation crosslink levels, known to accumulate during the ageing process, as measured by traditional biochemical methods. These results provide key evidence to support the application of Raman spectroscopy as a tool to detect chemical changes in the tendon, induced by ageing or pathology.

Compared to the younger tendons, aged tendons exhibited over three times higher CCD counts (combined fluorescence background and Raman spectrum) which positively correlated with collagen-linked fluorescence measured with a fluorometer (excitation 348 nm) following solubilization of tissue using enzymatic digestion. Previous studies on equine tendons showed a similar age-related increase in fluorescence level by both Raman spectroscopy [20] and biochemical methods [28]. Fluorescence background is often regarded as interference when interpreting the underlying Raman signals; however, as we have demonstrated here (and in [20]), relative fluorescence intensity alone can easily separate young and old tendons. Interestingly, by normalising the fluorescence–Raman combined spectra, both aged Achilles and anterior tibialis tendons demonstrated similar fluorescence background with a near-linear decline in intensities across the studied wavenumber region (800 to 1800 cm^−1^) while the younger tendons demonstrated an observable drop in the fluorescence level between 1100 cm^−1^ and 1400 cm^−1^. The different fluorescence background shapes are likely resulted from different types or quantities of fluorophores within tendons. In aged tendons, however, the greatly increased overall fluorescence intensity could mask these underlying subtle differences in fluorescence spectrum produced by native tendon compositions. AGE crosslinks can increase many times in aged tendons [6], while the total pyridinoline crosslinks, the predominant crosslink in adult tendons and also inherently fluorescent, usually remains stable [5,28] or even slightly decreases with age [6].

Glucosepane and pentosidine are two AGE crosslinks commonly increased in aged tendons. Glucosepane is non-fluorescent but present at considerably higher levels than the fluorescent pentosidine [5,6]. AGE crosslinks can alter tendon mechanical properties and affect cell-matrix interactions, resulting in reduced matrix repair ability after injury [3]. Raman spectral differences were identified between young and old tendons, and among these reported bands, several previously reported AGE crosslink-related bands [21,26,27] intensities were highly correlated with both the glucosepane and pentosidine quantity measured by biochemical techniques. Our results show that using Raman spectroscopy alone, with spectra normalised to the amide III band intensity, could rapidly and non-destructively provide a measure of the glycation crosslinks in tendon tissues with minimal sample preparation and processing.

Besides AGE crosslinks, the observed age-related difference in Raman spectra could be attributed to the highly anisotropic tendon sub-structure [22,29,30]. Van Gulick and colleagues demonstrated that aged rat tail tendon fibres are oriented more parallel to the fascicle long axis than younger tendons. This altered orientation was correlated with reduced amide I and CH_2_ bending bands and increased amide III band intensities in the measured Raman spectra [17], similar to our findings in aged human tendons. Interestingly, in our previous study [20] on equine superficial digital flexor tendons (SDFT), which has been confirmed with an altered fibre orientation in aged tendons [31], the Raman spectral differences between the young and old groups were only visible in CH_2_ bending and amide III bands, suggesting species differences or other influencing factors present. Further work is required to understand the relationship between human tendon sub-structural orientation and the Raman spectral changes. We were unable to conduct microscopic analysis of the tendon sub-structures due to the sample collection and preparation process. Future studies combining histological or microscopic analysis and Raman spectroscopy on human tendon orientation are now warranted based on our initial observations.

The compositional differences identified by Raman spectroscopy between the Achilles and tibialis anterior tendons allow separation by the PC1 loading. It has been reported that the human Achilles tendon has higher glycosaminoglycan and DNA content than the tibialis anterior tendon, with a similar total collagen content [6,32]. Interestingly, our previous study [20] demonstrated the most evident spectral differences between the equine SDFT and deep digital flexor tendon (DDFT) were present in the proline/hydroxyproline region (800–1000 cm^−1^) and CH_2_ bending band, without significant difference in amide I band [20]. Future comparison of tendons from different species should be made with caution since the vibrational mode of the specific tendon composition are yet unclear and often difficult to study in isolation.

Whilst we are able to make strong conclusions from our study it is pertinent to identify the limitations of this work. Firstly, the small sample size, which is due to the availability of human tendon tissue donors. Secondly, we can only report the correlation, not a causal relationship between Raman spectra and the glycation crosslinks quantities. Due to the complexity of biological tissue composition and the aggregated, combined spectral features, identifying a specific compositional change from the Raman spectrum alone is challenging. Lastly, we conducted multiple measurements on homogenised freeze-dried tissues to reduce the influence of tissue heterogeneity. However, it is likely that different compartments within the tendons (such as fascicles or the interfascicular matrix) have different compositions. Spectra from dried samples are also likely to have some differences to those from wet tissues since several Raman bands are sensitive to the hydration level of the tendon collagen [33]. Further studies are required on unprocessed hydrated tendon tissue to confirm our findings. In addition, future studies applying spectroscopic techniques, such as deconvolution of the amide bands, could elucidate the glycation-induced compositional or conformational changes to the tendon matrix.

## 4. Materials and Methods

### 4.1. Tendon Sample Collection

The Achilles tendon (n = 8, young:old = 4:4) and tibialis anterior tendon (n = 8, young:old = 4:4) were collected from amputated limbs of patients (young: 14–21 years, old: 65–87 years) at the Royal National Orthopaedic Hospital (Stanmore, UK). All patients gave consent for their tissue to be used for musculoskeletal related research (UCL/UCLH Biobank for Studying Health and Disease (HTA license number 12055) with Local R&D approval for this study (Ref: 11/0464)). Only tendons with no visual signs of injury or disease were selected. Tendons were harvested within 24 h of limb amputation, snap-frozen and stored at −80 °C. The samples were then freeze-dried, homogenised and underwent biochemical assays which were performed independently from Raman measurements [6].

### 4.2. Raman Spectroscopy Instrumentation

All samples (dried and homogenised) were analysed using a Renishaw inVia Raman spectrometer (Renishaw, Gloucestershire, UK) equipped with an 830 nm laser (300 mW at source). Ten Raman spectra from each sample were acquired (5 s exposure time, 10 accumulations) from randomly selected locations to reflect the heterogeneity of tendon composition. The spatial resolution was 2 × 2 µm using a 50× objective.

### 4.3. Raman Data Treatment and Analysis

In Raman spectroscopy, a highly fluorescent background in biological tissues is seen as interference and is usually removed at the first stage in the data analysis (e.g., by polynomial subtraction) [34]. We have developed a step-by-step analysis for tendon measurements that includes both combined spectra and fluorescence-removed Raman spectra for better differentiating age-related spectral differences, as described previously [20].

Collected raw spectra were truncated to wavenumber 800 cm^−1^ to 1800 cm^−1^ [11,20,35] and were then compared between groups for the discrepancy of fluorescence–Raman combined signal. Then, spectra were min-max normalised for visual inspection of different fluorescence background shapes. The fluorescence signal was then removed by an in-house MATLAB (The Mathworks, Inc., Natick, MA, USA) code using 6th order polynomial, and the subsequent Raman spectra were normalised to the peak amide III band intensity (between 1230 cm^−1^ and 1250 cm^−1^) [35]. Raman spectra were then pooled and underwent PCA (by Origin 2019, OriginLab Corporation, Northampton, MA, USA) to identify the segregation of tendon age and tendon types. Vector loadings of interested PC axes were plotted to visualise the variance in wavenumbers.

### 4.4. Biochemical Analysis

Pentosidine was quantified using reverse phase HPLC. Lyophilised tendon tissue (20 mg) was hydrolysed in 2 mL of 6M HCl for 24 h at 110 °C, and then dried under vacuum in a speed vac concentrator. The hydrolysate was re-suspended in D.I. H_2_O with 1% trifluoroacetic acid (TFA) at 15 mg/ml. 50 µL of sample was injected into the HPLC system (Shimadzu, Kyoto, Japan). Crosslinks were eluted using a Hypercarb column (150 × 4.6 mm, 7 µm i.d., Thermo Scientific, Waltham, MA, USA) at 25 °C with a flow rate of 1.0 mL/min. Mobile phase A was D.I. H_2_O with 1% TFA (*v*/*v*) and mobile phase B was 1% TFA in acetonitrile (*v*/*v*). The column was equilibrated with the starting ratio of A:B at 85:15 for 10 min and a gradient programme run from 85 to 45% mobile phase A over 20 min, followed by 5 min at 85% mobile phase A. The fluorometer was set to 335 nm excitation wavelength and 385 nm emission wavelength for the detection of pentosidine. After 25 min the column was washed with acetonitrile at 2 mL/min for 5 min before equilibration as above. Pentosidine concentration was calculated using a commercially available standard (Cayman Chemical supplied by Cambridge Bioscience Ltd., Surrey, UK). Collagen content was calculated by measuring the concentration of hydroxyproline, as previously described [36], in a 10 µL aliquot of the hydrolysate used for crosslink analysis. Glucosepane was quantified following sequential enzymatic digestion and LC-MS as described previously [6]. Collagen-linked fluorescence was quantified using a fluorometer set to 348 nm excitation wavelength and 457 nm emission wavelength following papain digestion of tissue as described previously [5].

### 4.5. Statistical Analysis

Pearson’s correlation coefficients were calculated (by SPSS v26, IBM, Armonk, NY, USA) between Raman band intensities and both pentosidine and glucosepane quantities. Independent *t*-tests were used to compare collagen-linked fluorescence and Raman band intensities between young and old groups, and between Achilles and tibialis tendons. The level of significance was set at 0.05, two-tailed.

## 5. Conclusions

In conclusion, we have demonstrated that Raman spectroscopy can differentiate minimally processed human Achilles and anterior tibialis tendon samples of different ages. The age-related spectral changes are highly correlated with differences in levels of the damaging glycation crosslinks, measured biochemically. Recent development of spatially offset Raman spectroscopy allows Raman spectra to be collect at depth within the tissue and through the skin. Taken together, these findings suggest that Raman spectroscopy has great potential as a tool to detect musculoskeletal tissue ageing or pathological changes of extracellular matrix chemistry.

## Figures and Tables

**Table 1:** Pearson’s correlation coefficients between Raman band intensities and biochemical assays of human Achilles and tibialis anterior tendons.

Raman Band	Pentosidine		Glucosepane	
	Achilles	Tibialis	Achilles	Tibialis
850 cm^−1^	0.01	−0.01	−0.09	−0.50
877 cm^−1^	0.75	0.82	0.66	0.79
918 cm^−1^	0.73	0.73	0.69	0.91
931 cm^−1^	0.34	0.72	0.17	0.81
951 cm^−1^	0.75	0.85	0.69	0.88
1002 cm^−1^	−0.68	−0.09	−0.71	−0.54
1130 cm^−1^	0.75	0.72	0.74	0.93
1270 cm^−1^	0.74	0.72	0.53	0.74
1293 cm^−1^	0.78	0.88	0.81	0.86
1324 cm^−1^	0.74	0.75	0.77	0.92
1450 cm^−1^	−0.83	0.04	0.77	−0.66
1660 cm^−1^	−0.80	0.21	−0.76	−0.55

**Table 2:** Raman band intensities between Achilles tendon (n = 80) and tibialis anterior tendon (n = 80).

Raman Band	Achilles Tendon	Tibialis Tendon	*p*-Value
825 cm^−1^	0.23 ± 0.05	0.38 ± 0.12	<0.001
850 cm^−1^	0.56 ± 0.03	0.47 ± 0.07	<0.001
877 cm^−1^	0.44 ± 0.05	0.51 ± 0.08	<0.001
931 cm^−1^	0.66 ± 0.04	0.59 ± 0.06	<0.001
1002 cm^−1^	0.66 ± 0.06	0.75 ± 0.11	<0.001
1030 cm^−1^	0.69 ± 0.03	0.77 ± 0.07	<0.001
1049 cm^−1^	0.52 ± 0.03	0.62 ± 0.05	<0.001
1082 cm^−1^	0.50 ± 0.05	0.57 ± 0.05	<0.001
1240 cm^−1^	0.97 ± 0.02	0.92 ± 0.03	<0.001
1270 cm^−1^	0.93 ± 0.05	0.98 ± 0.05	<0.001
1450 cm^−1^	1.11 ± 0.06	1.00 ± 0.07	<0.001
1660 cm^−1^	0.69 ± 0.03	0.63 ± 0.04	<0.001

## Data Availability

Not applicable.

## References

[1] Olson L.C., Redden J.T., Schwartz Z., Cohen D.J., Mcclure M.J. (2021). Advanced Glycation End-Products in Skeletal Muscle Aging. Bioengineering.

[2] Verzijl N., Bank R.A., TeKoppele J.M., DeGroot J. (2003). AGEing and osteoarthritis: A different perspective. Curr. Opin. Rheumatol..

[3] Snedeker J.G., Gautieri A. (2014). The role of collagen crosslinks in ageing and diabetes—The good, the bad, and the ugly. Muscles Ligaments Tendons J..

[4] Couppe C., Hansen P., Kongsgaard M., Kovanen V., Suetta C., Aagaard P., Kjaer M., Magnusson S.P. (2009). Mechanical properties and collagen cross-linking of the patellar tendon in old and young men. J. Appl. Physiol. (1985).

[5] Thorpe C.T., Streeter I., Pinchbeck G.L., Goodship A.E., Clegg P.D., Birch H.L. (2010). Aspartic acid racemization and collagen degradation markers reveal an accumulation of damage in tendon collagen that is enhanced with aging. J. Biol. Chem..

[6] Nash A., Notou M., Lopez-Clavijo A.F., Bozec L., de Leeuw N.H., Birch H.L. (2019). Glucosepane is associated with changes to structural and physical properties of collagen fibrils. Matrix Biol. Plus.

[7] Larkin P. (2011). Basic Principles. Infrared and Raman Spectroscopy.

[8] Larkin P. (2011). Origin of Group Frequencies. Infrared and Raman Spectroscopy.

[9] Larkin P. (2011). IR and Raman Spectra-Structure Correlations. Infrared and Raman Spectroscopy.

[10] Téllez S.C.A., Pereira L., Fávero P., Martin A.A. (2017). Confocal Raman Spectroscopic Analysis of the Changes in Type I Collagen Resulting from Amide I Glycation. Biomed. J. Sci. Tech. Res..

[11] Kerns J.G., Gikas P.D., Buckley K., Shepperd A., Birch H.L., McCarthy I., Miles J., Briggs T.W., Keen R., Parker A.W. (2014). Evidence from Raman spectroscopy of a putative link between inherent bone matrix chemistry and degenerative joint disease. Arthritis Rheumatol..

[12] Penteado S.C., Fogazza B.P., Carvalho Cda S., Arisawa E.A., Martins M.A., Martin A.A., Martinho Hda S. (2008). Diagnosis of degenerative lesions of supraspinatus rotator cuff tendons by Fourier transform-Raman spectroscopy. J. Biomed. Opt..

[13] Bergholt M.S., Serio A., Albro M.B. (2019). Raman Spectroscopy: Guiding Light for the Extracellular Matrix. Front. Bioeng. Biotechnol..

[14] Fields M., Spencer N., Dudhia J., McMillan P.F. (2017). Structural changes in cartilage and collagen studied by high temperature Raman spectroscopy. Biopolymers.

[15] Esmonde-White K. (2014). Raman spectroscopy of soft musculoskeletal tissues. Appl. Spectrosc..

[16] Rygula A., Majzner K., Marzec K.M., Kaczor A., Pilarczyk M., Baranska M. (2013). Raman spectroscopy of proteins: A review. J. Raman Spectrosc..

[17] Van Gulick L., Saby C., Morjani H., Beljebbar A. (2019). Age-related changes in molecular organization of type I collagen in tendon as probed by polarized SHG and Raman microspectroscopy. Sci. Rep..

[18] Talari A.C.S., Movasaghi Z., Rehman S., Rehman I.U. (2014). Raman Spectroscopy of Biological Tissues. Appl. Spectrosc. Rev..

[19] Martinez M.G., Bullock A.J., MacNeil S., Rehman I.U. (2019). Characterisation of structural changes in collagen with Raman spectroscopy. Appl. Spectrosc. Rev..

[20] Yin N.H., Parker A.W., Matousek P., Birch H.L. (2020). Detection of Age-Related Changes in Tendon Molecular Composition by Raman Spectroscopy-Potential for Rapid, Non-Invasive Assessment of Susceptibility to Injury. Int. J. Mol. Sci..

[21] Alsamad F., Brunel B., Vuiblet V., Gillery P., Jaisson S., Piot O. (2021). In depth investigation of collagen non-enzymatic glycation by Raman spectroscopy. Spectrochim. Acta A Mol. Biomol. Spectrosc..

[22] Galvis L., Dunlop J.W., Duda G., Fratzl P., Masic A. (2013). Polarized Raman anisotropic response of collagen in tendon: Towards 3D orientation mapping of collagen in tissues. PLoS ONE.

[23] Matousek P., Draper E.R., Goodship A.E., Clark I.P., Ronayne K.L., Parker A.W. (2006). Noninvasive Raman spectroscopy of human tissue in vivo. Appl. Spectrosc..

[24] Churchwell J.H., Sowoidnich K., Chan O., Goodship A.E., Parker A.W., Matousek P. (2019). Adaptive band target entropy minimization: Optimization for the decomposition of spatially offset Raman spectra of bone. J. Raman Spectrosc..

[25] Stone N., Baker R., Rogers K., Parker A.W., Matousek P. (2007). Subsurface probing of calcifications with spatially offset Raman spectroscopy (SORS): Future possibilities for the diagnosis of breast cancer. Analyst.

[26] Glenn J.V., Beattie J.R., Barrett L., Frizzell N., Thorpe S.R., Boulton M.E., McGarvey J.J., Stitt A.W. (2007). Confocal Raman microscopy can quantify advanced glycation end product (AGE) modifications in Bruch’s membrane leading to accurate, nondestructive prediction of ocular aging. FASEB J..

[27] Pereira L., Téllez Soto C.A., dos Santos L., Favero P.P., Martin A.A. (2015). Confocal Raman Spectroscopy as an Optical Sensor to Detect Advanced Glycation End Products of the Skin Dermis. Sens. Lett..

[28] Birch H.L., Bailey J.V., Bailey A.J., Goodship A.E. (1999). Age-related changes to the molecular and cellular components of equine flexor tendons. Equine Vet. J..

[29] Masic A., Bertinetti L., Schuetz R., Galvis L., Timofeeva N., Dunlop J.W., Seto J., Hartmann M.A., Fratzl P. (2011). Observations of multiscale, stress-induced changes of collagen orientation in tendon by polarized Raman spectroscopy. Biomacromolecules.

[30] Bonifacio A., Sergo V. (2010). Effects of sample orientation in Raman microspectroscopy of collagen fibers and their impact on the interpretation of the amide III band. Vib. Spectrosc..

[31] Thorpe C.T., Klemt C., Riley G.P., Birch H.L., Clegg P.D., Screen H.R. (2013). Helical sub-structures in energy-storing tendons provide a possible mechanism for efficient energy storage and return. Acta Biomater..

[32] Birch H., Smith T., Tasker T., Goodship A.E. Age Related Changes to Mechanical and Matrix Properties in Human Achilles Tendon. Proceedings of the 47th Annual Meeting, Orthopaedic Research Society.

[33] Masic A., Bertinetti L., Schuetz R., Chang S.W., Metzger T.H., Buehler M.J., Fratzl P. (2015). Osmotic pressure induced tensile forces in tendon collagen. Nat. Commun..

[34] Wei D., Chen S., Liu Q. (2015). Review of Fluorescence Suppression Techniques in Raman Spectroscopy. Appl. Spectrosc. Rev..

[35] Kerns J.G., Buckley K., Churchwell J., Parker A.W., Matousek P., Goodship A.E. (2016). Is the Collagen Primed for Mineralization in Specific Regions of the Turkey Tendon? An Investigation of the Protein-Mineral Interface Using Raman Spectroscopy. Anal. Chem..

[36] Birch H.L., Bailey A.J., Goodship A.E. (1998). Macroscopic ‘degeneration’ of equine superficial digital flexor tendon is accompanied by a change in extracellular matrix composition. Equine Vet. J..

